# A Deep Neural Network Framework for Dynamic Two-Handed Indian Sign Language Recognition in Hearing and Speech-Impaired Communities

**DOI:** 10.3390/s25123652

**Published:** 2025-06-11

**Authors:** Vaidhya Govindharajalu Kaliyaperumal, Paavai Anand Gopalan

**Affiliations:** Department of Computer Science and Engineering, SRM Institute of Science and Technology, Vadapalani, Chennai 600026, India; pavai_gops@yahoo.co.in

**Keywords:** vision transformers (ViT), sign language (SL), human–computer interaction (HCI), tuna swarm optimization (TSO), enhanced convolutional transformer (ECT), convolutional neural network (CNN), indian sign language (ISL)

## Abstract

Language is that kind of expression by which effective communication with another can be well expressed. One may consider such as a connecting bridge for bridging communication gaps for the hearing- and speech-impaired, even though it remains as an advanced method for hand gesture expression along with identification through the various different unidentified signals to configure their palms. This challenge can be met with a novel Enhanced Convolutional Transformer with Adaptive Tuna Swarm Optimization (ECT-ATSO) recognition framework proposed for double-handed sign language. In order to improve both model generalization and image quality, preprocessing is applied to images prior to prediction, and the proposed dataset is organized to handle multiple dynamic words. Feature graining is employed to obtain local features, and the ViT transformer architecture is then utilized to capture global features from the preprocessed images. After concatenation, this generates a feature map that is then divided into various words using an Inverted Residual Feed-Forward Network (IRFFN). Using the Tuna Swarm Optimization (TSO) algorithm in its enhanced form, the provided Enhanced Convolutional Transformer (ECT) model is optimally tuned to handle the problem dimensions with convergence problem parameters. In order to solve local optimization constraints when adjusting the position for the tuna update process, a mutation operator was introduced. The dataset visualization that demonstrates the best effectiveness compared to alternative cutting-edge methods, recognition accuracy, and convergences serves as a means to measure performance of this suggested framework.

## 1. Introduction

People communicate with one another in a significant way to express their basic needs and ideas. Hearing and speech-impaired people rely on Sign Language (SL) as their primary means of communication. According to WHO reports, 466 million people worldwide are estimated to have hearing impairment [1]. The hearing-impaired communities are the ones who use SL the most, and it facilitates communication between them and other regular people. SL is the intricate collection of words and phrases conveyed through facial expressions, hand and finger gestures, and body language [2]. According to the reports, 70 million people worldwide use SL for communication.

For millions of Indians, Indian Sign Language (ISL) is the most important of the country’s many national sign languages. Automatically recognizing ISL, particularly dynamic two-handed gestures, is still a difficult task. Continuous hand movements, shifting shapes, and complex spatiotemporal patterns are all features of dynamic signs that make machine interpretation more difficult. Although deep learning-based vision-based recognition techniques have shown promise, current approaches frequently have trouble accurately modeling the long-range spatial dependencies and fine-grained local details present in dynamic signs. We suggest a novel hybrid framework that combines Adaptive Tuna Swarm Optimization (ATSO) and an Enhanced Convolutional Transformer (ECT) in order to address these issues. The ECT-ATSO model successfully strikes a balance between contextual and detailed gesture understanding by utilizing Vision Transformers to capture global dependencies and CNNs for local feature extraction. Additionally, the ATSO algorithm solves convergence problems common in challenging high-dimensional recognition tasks and guarantees effective hyperparameter tuning.

SL includes both its grammar and syntax, and different gestures have a different meaning than in other languages. For the hearing and speech-impaired, several SLs were created. Only a few of these SLs are recognized by law. Each country and the subcontinent have their own SL, and each country has created its own SL [3,4]. Static signs consist of a fixed hand posture, while dynamic signs consist of a variety of facial expressions and hand movements [5]. Hearing and speech-impaired people need SL recognition in order to access everyday necessities like general information, medical care, social commitment, education, and more. Both device-based and vision-based methods are used in the gesture recognition process. To identify the pattern, vision-based SL recognition is typically used [6,7].

The detection of SL in videos is more difficult, though, because it must deal with variations in motion and speed, small fingers, and shifting lighting. To accurately identify SLs in the hearing and speech-impaired communities, some automatic SL recognition techniques must be created. In SL recognition systems, identifying continuous sign words with both single and double hands is the main task. Human-Computer Interaction (HCI) techniques have been developed by numerous researchers for the automatic recognition of gesture, activity, and gait [8].

These methods enable interactions between humans and robots as well as various detection applications. Additionally, deep learning techniques like CNN and Recurrent Neural Network (RNN) have been modified for automatic SL recognition among wearable sensor-based application approaches. Better movement tracking and SL word recognition performance outcomes are offered by the aforementioned methods. Even though the aforementioned methods produce better results, they have drawbacks like unnatural use, background complexity, movement flexibility, and calibration adjustment. Furthermore, SL words with the identical motion, orientation, and pattern are not recognized by these methods [9,10]. This study presents a novel Enhanced Convolutional Transformer with Adaptive Tuna Swarm Optimization (ECT-ATSO) method to identify double-handed Sign Language for people who are deaf or dumb in order to address these issues.

The following are the main contributions to the recognition of double-handed sign language:

Goal: This study uses an Adaptive Tuna Swarm Optimization (ATSO) algorithm based on an Enhanced Convolutional Transformer (ECT) with a dynamic word to identify double-handed sign language for the hearing-impaired people. The proposed dataset is created for this purpose using an ISL-based ISL-CLRT dataset.

Data Preparation: To improve visual fidelity and the suggested framework’s capacity for generalization, methods like augmentation, normalization, and image denoising are used to preprocess the data that are gathered from the generated proposed dataset.

Graining and extraction of features: It is assumed that the preprocessed images’ features are grained using multiple convolutional layers. To improve prediction accuracy and performance, the CViT model is used for feature extraction, extracting both local as well as global features.

Classification and tuning: Various dynamic words are used to classify the feature map that was obtained from the concatenated output. The transformer-based classification model’s parameters are adjusted by the TSO algorithm, and the model is rescued from the local optimum problem by means of the mutation operation. Overall, the convergence issue and higher-dimensional problem are handled by the ATSO algorithm. Evaluation of performance: Accuracy and validation of the provided dataset are used to assess the performance of this suggested model during convergence analysis. At the 100th iteration, the suggested ECT-ATSO algorithm converged to a value of 0.987 and achieved a recognition accuracy of 98.69%.

The remaining research sections are arranged as follows: The different methods that have been used in double-handed SL recognition are reviewed in Section 2. The suggested method’s operational flow is described in Section 3. The implementation details and empirical findings gathered by the suggested method are presented in Section 4. This research is finally concluded with future directions in Section 5.

## 2. Literature Survey

In order to recognize double-handed Sign Language, numerous analyses have been carried out using various methods, such as Transformer-based, Deep Learning, Machine Learning, and Optimization-based methods. Few studies were conducted to identify the dual-handed Sign Language used by people with hearing and speech impairments. The development of an efficient model for the identification of dual hand signs was facilitated by the strengths and weaknesses of such analyses.

### 2.1. Recognition of Sign Language Using Machine Learning

A MultiLayer-based Kernel Extreme Learning Machine (ML-KELM) approach was presented by Katilmi and Karakuzu [11] for dual-hand dynamic Turkish Sign Language (TSL) word recognition. Reducing the dimensionality of certain features improved classification performance. For double-handed dynamic TSL recognition, Katilmi and Karakuzu [12] employed the Meta-ELM technique. Time Frequency Domain Feature Extraction (TFDFE) was used to regularize the data.

Using various Machine Learning (ML) techniques, Alrubayi et al. [13] presented a recognition system for Malaysian Sign Language (MSL). The highest recognition and accuracy rates were attained by K-nearest Neighbor (KNN), Random Forest (RF), and Artificial Neural Network (ANN) techniques out of all the machine learning techniques.

Tamiru et al. [14] used machine learning approaches such as Support Vector Machine (SVM) and ANN to identify the signs of Amharic Sign Language (AMSL). Thirty-four hand sign features were chosen for this study, and the SVM model outperformed the ANN model. The communication gap between those with hearing impairments was lessened by the model.

A reliable automated sign language recognition system for both one-handed and dual-handed sign language recognition was presented by Roy et al. [15]. Camshift tracking was used to obtain the hand trajectories, and the Hidden Markov Model (HMM) was used for detecting Sign Language. Using the new hand trajectories and skin region features improved the system’s classification performance.

### 2.2. Deep Learning Applied for Sign Language Recognition

ISL can be automatically recognized with two hands using Deep Learning models, according to Sreemathy et al. [16]. The Histogram of Gradient (HOG) was utilized to extract features for sign language recognition, and to increase accuracy, a recurrent local contrast normalization model was used. The model that demonstrated equilibrium among the input patterns was trained using the Back-Propagation Network (BPN).

An SVM-based CNN model for Indian Sign Language alphabet, digit recognition was demonstrated by Kotach et al. [17]. After extracting the Speeding Up Robust Features (SURF)-provided frame, histograms were created to represent the sign language. Using the Bag of Visual Words (BOVW), predictions were made and histograms were formed.

Lee et al. [18] used the LSTM with RNN and KNN to handle the incoming input patterns in order to recognize American Sign Language (ASL). Monochromatic infrared cameras and infrared LEDs were used to obtain features such as the angles of the fingers, sphere radius, and finger-to-finger distance. When extracting the thumb features, the model’s robustness and low sensitivity were demonstrated by the best performance.

By improving the model’s performance and efficiency, Shin et al. [19] introduced a Transformer-based CNN (T-CNN) model for Korean Sign Language (KSL) classification. Both local and long-term dependency features can be handled by this hybrid model for the subsequent classification procedure. Twenty classes from the KSL dataset were used, and the classification process performed better.

A 3D CNN was proposed by Sharma and Kumar [20] to enable recognition of ASL from video sequences. In order to lower the communication barrier, Kothadiya et al. [21] created an LSTM-based Gated Recurrent Unit (LSTM-GRU) model for sign language identification. Isolated ISL (IISL) video frames were used to obtain the sign language data, and the method performed better when helping the hearing and speech-impaired.

In order to break down the difficulty in communication between deaf individuals and non-deaf individuals, Nandi et al. [22] illustrated a CNN model that can identify the ISL alphabet. A feature map was created using the particular features in the input images, and by modifying the learning rate and weights of the feature representation, the Stochastic Gradient Descent (SGD) optimizer was utilized to lower error rates, and maximize accuracy.

### 2.3. Recognition of Sign Language Using Transformers

To refine text-to-sign interpretation performance, Chaudhary et al. [23] presented SignNet II, a sign language system supporting two-way communication interpretation system powered by transformers. A GRU-based Relative Sign Transformer (GRU-RST) framework was presented by Aloysius et al. [24] for continuous conversion and identification of sign language. The RWTH-PHOENIX-2014T dataset was used for testing, and it produced translations of higher quality than typical spoken text.

A Twin Delayed Deep Reinforcement Memory Network (TDDRMN) architecture was presented by Karthick et al. [25] for word-level sign language prediction. Since the feature representation was in vector form, the Linear Embedded Hessian Component Analysis (LEHCA), and its probability was computed for feature extraction.

A Minimum Redundancy and Maximum Relevance-based Particle Swarm Optimization (mRMR-PSO) approach was presented by Bansal et al. [26] for the recognition of sign language. Three different sign languages were identified using publicly accessible datasets, and the classification accuracy was improved through the Multi-Class SVM model, which reduced the number of features. In order to identify sign languages, Fregoso et al. [27] presented a PSO-CNN method. This CNN model gathers object features and key information to improve efficacy and lower computation costs.

### 2.4. Research Gap

Deep learning techniques for sign language recognition have made significant strides, but they still have significant drawbacks when it comes to handling dynamic two-handed gestures, particularly in the context of Indian Sign Language (ISL). The majority of earlier works ignore the distinct structural and grammatical differences of ISL in favor of either concentrating on static gestures or adapting them for American Sign Language (ASL). Moreover, conventional CNN- or RNN-based models frequently have trouble capturing both long-range dependencies and local spatial features, which are crucial for identifying continuous, dynamic gestures. The majority of earlier research has ignored the distinct grammatical and spatial features of Indian Sign Language (ISL) in favor of either static gestures or American Sign Language (ASL).

Additionally, the long-range dependencies and local spatial features needed for continuous gesture recognition are frequently not captured simultaneously by traditional CNN and RNN models. Seldom are optimization techniques to enhance model generalization, robustness, and convergence speed investigated. These drawbacks show how urgently an ISL-centric, specialized recognition framework is needed in order to accurately, adaptively, and computationally efficiently model complex dynamic gestures. Table 1 provides an overview of the research gaps that have been identified and our suggested solutions.

### 2.5. Advances in Sign Language

#### Recognition and Translation

Applying deep learning and sequence modeling techniques has led to notable advancements in the field of sign language translation and recognition in recent years. The Hierarchical Recurrent Deep Fusion framework, which uses adaptive clip summarization for sign language translation tasks, is one example of a hierarchical recurrent architecture that has been proposed to improve the temporal modeling of sequential gestures [1]. Some methods have used sign back-translation with monolingual data to enhance translation performance in low-resource settings in order to overcome the lack of parallel sign language datasets [2]. In sign language translation, hierarchical LSTM networks have also been used to model intricate sequential dependencies, leading to appreciable gains in recognition accuracy and fluency [3].

Transformer-based models have been used more recently for both translation and recognition of sign language; systems such as Sign Language Transformers provide end-to-end translation and recognition capabilities in a single architecture [4]. By successfully extracting spatiotemporal patterns from continuous video sequences, time series neural networks have also shown promise in real-time sign language translation [5]. Additionally, to incorporate visual, spatial, and temporal cues, graph-based multimodal sequential embedding frameworks have been developed, offering enhanced contextual representations for sign language translation [6].

These modern methods demonstrate the increasing popularity of transformer-based, multi-modal, and hierarchical models for sign language processing, which complement and match the goals of our suggested ECT-ATSO framework for dynamic dual-handed sign language recognition.

## 3. Proposed Work

**Figure 1:**
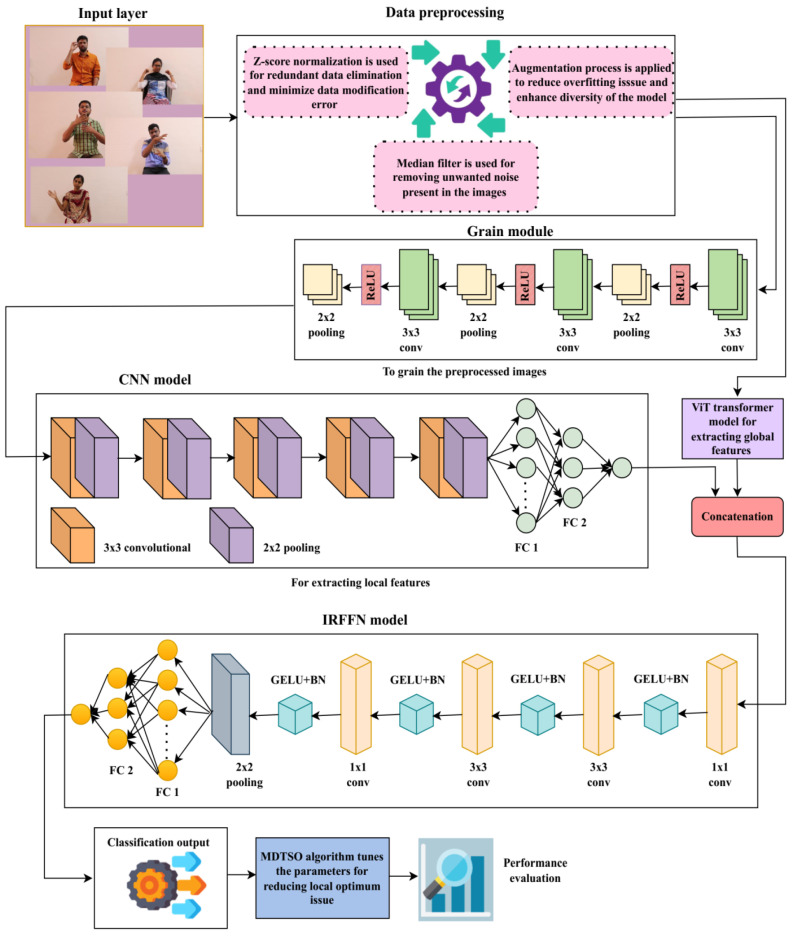
Work flow of the proposed method.

Figure 1 depicts the process of the suggested double-handed SL recognition method. The proposed dataset is derived from the ISL-CSLTR dataset, which contains the input images. To achieve the best visual fidelity and extension capability, the proposed dataset’s images are first sent to the preprocessing step. The noise removal, normalization, and image augmentation procedures are all part of the data preprocessing. A statistical median filter is used to get rid of unwanted distortions from the image during denoising process. By removing redundant and unstructured data, the Z-score normalization process transforms the data for consistency. By increasing the range of data used for training, the data augmentation reduces the problem of overfitting. Following pretreatment, preprocessed images undergo grain modeling for texture enhancement.

The CViT framework, which consists of two blocks—the CNN and ViT transformer—is then used for feature extraction. Only local characteristics of the grained model are retrieved by the CNN model, and only global features are extracted by the ViT transformer. The feature representation is created through the means of concatenating these local as well as global features integrated through a concatenation block. The feature map that has been created is then sent to the classification stage. The stage of classification provides results from the multi-class classification, while the IRFFN model creates the variable sign language terms derived based on the activation map at this point. The TSO algorithm is implemented to overcome the local optimum challenge and fine-tune CViT model parameters during the training phase. To address this issue, the TSO algorithm uses the mutation operator, and the ATSO algorithm is added to achieve the best possible output.

### 3.1. Data Processing

We use the ISL-CSLTR dataset, which is accessible to the public, as the base dataset for this investigation. The enhanced dataset that results from applying preprocessing techniques like denoising, normalization, and augmentation is referred to as the “proposed dataset” throughout the manuscript.

Data preprocessing improves quality and generalization capacity of raw data by transforming it into a readable format. Image denoising, normalization, and data augmentation procedures are used in data processing. The proposed dataset is used to gather images that will be used as input during preprocessing.

#### 3.1.1. Image Denoising

The process of removing unwanted noise from images is known as image denoising [28]. The most popular filtering method for removing noise from photos and safeguarding edges is the median filter. The intensity values at a pixel’s location is how the midpoint filter spreads the pixel’s evaluation. The rank command statistic, which indicates the outcome for the primary flow of pixel values, is another name for the median. The definition of the statistical median filter is

(1) $$l^{\prime }\left(a,b\right)=\underset{\left(u,v\right)\in K_{ab}}{med}\left\{w\left(u,v\right)\right\},$$

where $w\left(u,v\right)$ denotes the noisy image, med corresponds to the median, $l^{\prime }\left(a,b\right)$ represents the filtered image, and $K_{ab}$ indicates the filter mask.

#### 3.1.2. Normalization

The process of reorganizing data by eliminating redundant and unstructured information is known as normalization [29]. The most widely used normalization technique is Z-score normalization, which is measured with respect to the mean and standard deviation of the data. This workflow is particularly useful in cases where the highest, actual, and lowest data values are uncertain. The Z-score method is defined by the following formula:

(2) $$S_{nw}=\frac{S-\eta }{\lambda }=\frac{S-mn\left(S\right)}{sdv\left(S\right)},$$

Here, mn signifies the mean, whereas η stands for the aggregate mean, $S_{nw}$ represents the new value derived from normalized results, sdv indicates the standard deviation, λ denotes the standard deviation value, and S signifies the former value.

#### 3.1.3. Augmentation

Artificial process of creating new data points from preexisting data is known as data augmentation. Data augmentation increases the training data’s diversity and lessens the issue of overfitting.

### 3.2. Convolution—Enhanced Vision Transformer (CViT)

The preprocessed image is first grained using the grain model as part of the feature extraction process [30]. This grain module uses three convolutional blocks, each of which consists of a 2 × 2 max pooling layer, a Rectified Linear Unit (ReLU) activation function, and a 3 × 3 convolution. The CViT transformer model is used to extract hand position, hand shape, and hand movement features.

The CNN architecture, represented by the symbol $Conv\left(Y\right)$, is used to extract the local details extracted from the grained system. To extract local features, this CNN model uses a variety of convolutional, pooling, and fully connected layers. Each image’s features are learned by the convolutional block by means of iterative cycles that produce a generated feature representation. This feature extraction model uses multiple pooling layers to reduce the feature map’s resolution by integrating similar features. By reducing the feature map’s dimensions, this layer cuts down on training time and avoids overfitting. By utilizing the activation function, the suggested model’s learning speed and classification performance are improved. Reducing expenses, cutting down on computation time, and avoiding overfitting issues are the primary benefits of employing the CNN model. The CNN architecture’s feature map is represented as follows:

(3) $$F_{Conv}=Conv\left(Y\right),$$

The preprocessed images are used as input for a transformer model’s patch embedding in order to extract global features. The input images $Y\in I^{H\times \omega \times C}$ are divided into patches of uniform size during this patch embedding step. Here, the terms such as H, ω, and C and height, width, and the respective channel numbers. The following formula is applied to determine how many patches are used in the transformer model:

(4) $$M=\frac{H\times \omega }{R^{2}},$$

where *R* is the specified image patch resolution. This model also incorporates a position embedding to capture positional information, and it assumes that the patch embedding will result in a linear projection. These position embedding (Position) and linear projection (Linear) have been combined for the learning process and are inferred by:

(5) $$P\left(Y\right)=Conv\left(Linear,\;Position\right),$$

The Multi-Layer Perceptron (MLP) and the Multi-Head Attention (MHA) layers serve as the foundation for the transformer encoder block’s design. The MHA layer converts the input into Query(), Key(), and Value(). The three computational matrices are expressed as follows after being computed using their respective weights:

(6) $$Q=P\left(Y\right)\times \varpi_{Q},$$

(7) $$K=P\left(Y\right)\times \varpi_{K},$$

(8) $$V=P\left(Y\right)\times \varpi_{V},$$

Referring to the above equation, the terms $\varpi_{Q}$, $\varpi_{K}$, and $\varpi_{V}$ are defined as the weights corresponding to the query, key, and value. Transforming the input feature to ensure that enhances accuracy and computational time is referred to as the norm. The MHA layer receives the outcome of this layer. The MHA layer’s predicted output is assessed using the following equation:

(9) $$A=\mathrm{softmax}\left(\frac{Q\;K^{T}}{\sqrt{\delta_{K}}}\right)\times V,$$

The representation of query, key, and value dimensions is given by $\delta_{Q}$, $\delta_{K}$, and $\delta_{V}$, respectively. The MLP and the MHA layers both use the following normalization function:

(10) $$Norm_{M}=Norm\left(Y+M\left(Y\right)\right),$$

(11) $$Norm_{MLP}=Norm\left(Y+MLP\left(Y\right)\right),$$

A Gaussian Error Linear Unit (GELU) incorporating two fully connected layers is used to create the MLP layer, which is the final result of the transformer encoder model. The transformer encoder’s final output is obtained as

(12) $$F_{Trans}=Trans\left(Y\right),$$

Using a concatenation block, global and local features are integrated to create the feature map, which is provided by

(13) $$X=Concat\left(F_{Conv},\;F_{Trans}\right),$$

**Figure 2:**
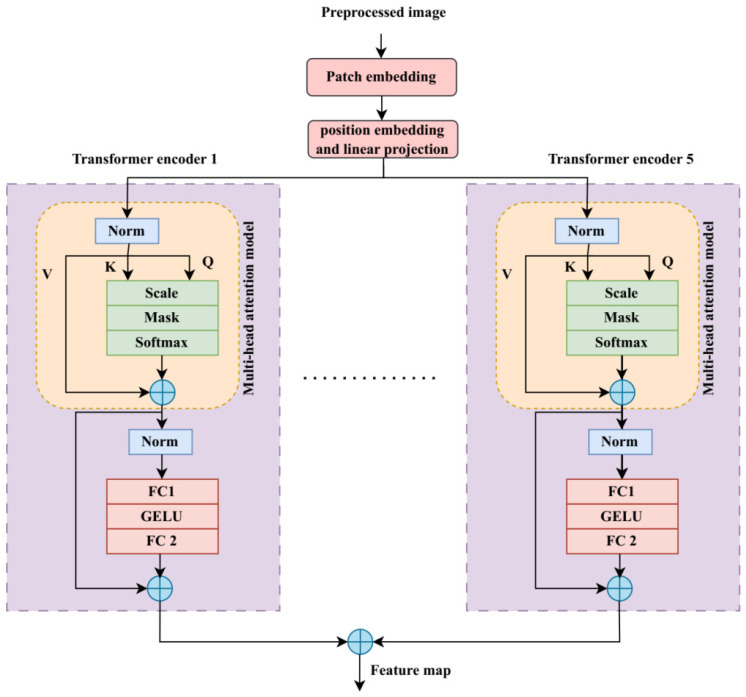
Diagram of the Vision Transformer (ViT).

Figure 2 illustrates the ViT transformer’s structure.

Local features in this work are spatial details that are limited to small receptive fields and are efficiently captured by convolutional filters, such as finger edges, hand contours, and texture patterns. Conversely, long-range dependencies, like the relative position and motion of both hands and coordinated hand face movements, are a feature of global gestures. Through their self-attention mechanism, Vision Transformers (ViTs) can model these global relationships, allowing the framework to interpret semantically related but spatially distant gesture components within a dynamic sign.

### 3.3. Network Model with Inverted Residual Feed-Forward (NM-IRFF)

The feed-forward network has been modified to use a classifier. Convolutional layers, GELU activation functions, batch normalization, average pooling layers, and fully connected layers are all used in its construction. Features’ dimensions are decreased at each convolutional layer, and the output of an average pooling layer is predicted using element-wise convolution.

(14) $$F_{Avg}=Avg\left(Conv\left(Conv\left(Conv\left(Y\right)\right)\right)\right),$$

(15) $$F\left(X\right)=F\left(F_{Avg}\right),$$

The results of a fully connected layer and an average pooling layer are shown by $F_{Avg}$ and $F\left(X\right)$. In the labeling stage, the sign language recognition model’s multi-class classification output was obtained.

### 3.4. Enhanced Convolutional Transformer with Adaptive Tuna Swarm Optimization (ECT-ATSO) Algorithm

A CNN-based ViT transformer’s parameter is adjusted utilizing the ECT-ATSO method, which manages multidimensional challenges and optimization constraints.

#### 3.4.1. TSO Algorithm

Thunnini is the scientific name for the tuna, an ocean predator fish. Tunas consume both mid water and surface fish. Tunas’ capacity to swim continuously is both exceptional and effective. Tunas use the group travel strategy for predation. To locate and pursue their prey, tunas use their intelligence. Additionally, tunas have evolved a variety of clever and effective foraging strategies, including parabolic and spiral foraging. To reposition their prey through the shallow water and attack with ease, tunas use the spiral formation they create while swimming as part of their foraging strategy.

- Initialization

The initial populations on the search space are created at random by the TSO algorithm to start the optimization process.

(16) $$G_{e}^{idv}=ℜ\cdot \left(Upp_{bo}-Low_{bo}\right)+Low_{bo},\;\;\;e=1,2,\ldots ,num_{po},$$

The search space’s minimum and maximum are marked by $Low_{bo}$ and $Upp_{bo}$, the random vector within the range of $\left[0,1\right]$ is expressed as ℜ, the total tuna populations are depicted as $num_{po}$, and the $e^{th}$ initial individual is denoted by $G_{e}^{idv}$.

- Spiral Foraging

When small schooling fish encounter carnivores, they all create a dense pattern that constantly changes their swimming direction. As a result, the carnivores find it difficult to contain the prey. In order to pursue their prey, the tuna group creates a close spiral structure. A large number of fish lack a strong sense of direction. As the gathering of fish swims vigorously along a designated route, adjacent fish alter their path one after another, form the largest group with a shared goal, and start hunting. Every tuna pursues the previous fish, and the schools of tuna communicate with each other. Therefore, the tuna in the area is informed. The mathematical expression for the spiral foraging scheme is

(17) $$G_{e}^{f+1}=\left\{\begin{array}{cc} \gamma_{1}\cdot \left(G_{best}^{f}+\mu \cdot \left|G_{best}^{f}-G_{e}^{f}\right|\right)+\gamma_{2}\cdot G_{e}^{f}, & e=1\\ \gamma_{1}\cdot \left(G_{best}^{f}+\mu \cdot \left|G_{best}^{f}-G_{e}^{f}\right|\right)+\gamma_{2}\cdot G_{e-1}^{f}, & e=2,3,\ldots ,num_{po} \end{array}\right.,$$

(18) $$\gamma_{1}=j+\left(1-j\right)\cdot \frac{f}{f_{\max}},$$

(19) $$\gamma_{2}=\left(1-j\right)-\left(1-j\right)\cdot \frac{f}{f_{\max}},$$

(20) $$\mu =\exp\left(qr\right)\cdot \cos\left(2\pi q\right),$$

(21) $$r=\exp\left(3\cos\left(\left(\left(f_{\max}+1/f\right)-1\right)\pi \right)\right),$$

where $f_{\max}$ represents the iteration limit, the given constant is indicated by j, the $e^{th}$ individual for iteration f+1 is denoted by $G_{e}^{f+1}$, the weight coefficients are indicated by $\gamma_{1}$ and $\gamma_{2}$, a randomly selected number between $\left[0,1\right]$ is expressed as q, the current best individual is denoted by $G_{best}^{f}$, and the current iteration is represented by f. Each tuna has the ability to exploit the space around its prey. Additionally, the search space generates the random coordinate for spiral foraging, which helps each individual search a larger area and creates the TSO with the ability to explore the entire world. It is described as

(22) $$G_{e}^{f+1}=\left\{\begin{array}{cc} \gamma_{1}\cdot \left(G_{ℜ}^{f}+\mu \cdot \left|G_{ℜ}^{f}-G_{e}^{f}\right|\right)+\gamma_{2}\cdot G_{e}^{f}, & e=1\\ \gamma_{1}\cdot \left(G_{ℜ}^{f}+\mu \cdot \left|G_{ℜ}^{f}-G_{e}^{f}\right|\right)+\gamma_{2}\cdot G_{e-1}^{f}, & e=2,3,\ldots ,num_{po} \end{array}\right.,$$

where $G_{ℜ}^{f}$ shows the stochastically determined reference point inside the feasible region. When the iteration is increased, the TSO moves the navigational points in the spiral foraging pattern from the random individuals to the best individuals. The derived final expression for the spiral search strategy scheme can be expressed as:

(23) $$G_{e}^{f+1}=\left\{\begin{array}{l} \left\{\begin{array}{l} \gamma_{1}\cdot \left(G_{ℜ}^{f}+\mu \cdot \left|G_{ℜ}^{f}-G_{e}^{f}\right|\right)+\gamma_{2}\cdot G_{e}^{f},\;e=1\;\\ \gamma_{1}\cdot \left(G_{ℜ}^{f}+\mu \cdot \left|G_{ℜ}^{f}-G_{e}^{f}\right|\right)+\gamma_{2}\cdot G_{e-1}^{f},\;e=2,3,\ldots ,num_{po} \end{array}\right.,\;if\;ℜ<\frac{f}{f_{\max}}\\ \left\{\begin{array}{l} \gamma_{1}\cdot \left(G_{best}^{f}+\mu \cdot \left|G_{best}^{f}-G_{e}^{f}\right|\right)+\gamma_{2}\cdot G_{e}^{f},\;e=1\;\\ \gamma_{1}\cdot \left(G_{best}^{f}+\mu \cdot \left|G_{best}^{f}-G_{e}^{f}\right|\right)+\gamma_{2}\cdot G_{e-1}^{f},\;e=2,3,\ldots ,num_{po} \end{array}\right.,\;if\;ℜ\geq \frac{f}{f_{\max}} \end{array}\right.,$$

- Parabolic Foraging

The tuna develop the parabolic structure from the designated point, which in this case is food. In addition, the tuna explore their surroundings. Therefore, both of these techniques are used concurrently, and a 50% selection probability is applied to both techniques. The following equation displays the mathematical expression:

(24) $$\begin{array}{l} G_{e}^{f+1}=\left\{\begin{array}{l} G_{best}^{f}+ℜ\cdot \left(G_{best}^{f}-G_{e}^{f}\right)+XC\cdot a^{2}\cdot \left(G_{best}^{f}-G_{e}^{f}\right),\;\;\;if\;ℜ<0.5\\ XC\cdot a^{2}\cdot G_{e}^{f},\;\;\;\;\;\;\;\;\;\;\;\;\;\;\;\;\;\;\;\;\;\;\;\;\;\;\;\;\;\;\;\;\;\;\;\;\;\;\;\;\;\;\;\;\;\;if\;ℜ\geq 0.5 \end{array}\right. \end{array},$$

(25) $$a={\left(1-\frac{f}{f_{\max}}\right)}^{\left(f/f_{\max}\right)}$$

The random number in this instance falls within the range of $\left[1,-1\right]$ indicated by XC. Until the termination criterion is satisfied, each TSO will be updated and continuously computed during the processing of the entire optimization. Additionally, the best individual and the corresponding fitness value are identified.

#### 3.4.2. Mutation Operation

Once the tuna positions are updated at each iteration, the mutation operator is used in the TSO algorithm to prevent the causes of local optimum problems. The mutation operator can be expressed mathematically as follows:

(26) $$G_{f+1}=G_{f}+\omega \times \left(Upp_{bo}-Low_{bo}\right)\times ℜ,$$

The scale factor is shown here by ω. The pseudo-code for the Enhanced Convolutional Transformer with Adaptive Tuna Swarm Optimization (ECT-ATSO) algorithm is given below (Algorithm 1).

**Algorithm 1.** Pseudocode for ECT-ATSO1. Begin
2. Set Up Parameters
3. While $\left(f<f_{\max}\right)$
Use Equation (27) to calculate the fitness value.
              For (Iteration)
                          Revise $\gamma_{1},\;\gamma_{2},\;a$
If (ℜ<m)                                                     //m=0.05
                                        Revise $G_{e}^{f+1}$ utilizing Equation (15)
                                Else If (ℜ≥m)
                                          If (ℜ<0.5)
                                                  If $\left(f/f_{\max}<ℜ\right)$
                                                      Update $G_{e}^{f+1}$ utilizing Equation (21)
                                                  Else If $\left(f/f_{\max}\geq ℜ\right)$
                                                  Update $G_{e}^{f+1}$ utilizing Equation (16)
                                                        Else If (ℜ≥0.5)
                                                                Update $G_{e}^{f+1}$ utilizing Equation (23)
                                              End If
                      End If
              End For
                Apply mutation operator using Equation (25)
f=f+1
    End of While
Provide the best possible solution.
4. End.


### 3.5. Procedure for Training and Optimizing

The CNN-based ViT transformers’ hyperparameters are changed during training in order to increase accuracy by utilizing the ATSO algorithm. In order to tackle the problem of disparities in classes, the loss function is used. The most widely used loss function for handling multiple class distinctions is the categorical loss function based on cross-entropy. The categorical cross-entropy loss ($CA_{CEL}$) is computed as

(27) $$CA_{CEL}\left(g,h\right)=-\frac{1}{K}\sum_{l=1}^{K}\sum_{d=1}^{D}g_{l,d}\cdot \log\left(h_{l,d}\right)$$

where D indicates distinct class count, K specifies the sample size, $g_{l,d}$ represents the actual class probability, and $h_{l,d}$ represents the predicted values matrix for each class.

The optimal solution is assessed using a fitness function in order to get around the optimization issues in the suggested method. This function is derived from the recognition accuracy shown in Equation (27), where better performance is indicated by a higher fitness value. The steps listed below describe the methodology used in this study:

- Step 1: Use the proposed dataset to collect data.
- Step 2: Preprocess the data to improve the generalization capacity related to the model and the quality of images. Equations (1) and (2) are utilized to accomplish this.
- Step 3: Give the pictures feature graining.
- Step 4: To increase prediction accuracy, extract both local and global features. Equation (12) creates a feature map for additional analysis based on recognition accuracy.
- Step 5: Use Equation (14) to categorize the extracted features into various dynamic words for double-handed sign language.
- Step 6: Adjust hyperparameters to address high-dimensionality problems and convergence issues. To avoid local optima and improve individual position updates, the optimization algorithm incorporates a mutation operator.
- Step 7: Consider the most ideal answer.

## 4. Assessments and Results

The experimental results of the suggested double-handed SL recognition method are covered in this section. A thorough explanation of the implementation-related topics, including hardware, software specifications, and hyperparameter tuning, opens this section. The performance metrics and datasets used in this study are then described. The performance evaluation and comparative analysis results are finally covered in this section in tabular and graphical form.

### 4.1. Experimental Setup

An Intel Corei3-1005G1 CPU running at 1.2 GHz, 64-bit OS, 8GB RAM, and Windows 11 was used to test the experimental evaluations. The Python 3.11 programming language was used to implement the suggested approach.

### 4.2. Hyperparameter Configurations

To choose the optimal hyperparameters for improved performance, the hyperparameter tuning process is utilized. The suggested method performs better with the ideal hyperparameters chosen; Table 2 displays these hyperparameters.

### 4.3. Dataset Specification

This paper establishes communication between the hearing and speech-impaired using the sentence-level fully labelled ISL-CSLTRL dataset. The hand positions, gaze, eye, and mouth movements are examples of the manual and nonmanual aspects of sign language. Four student volunteers and two native signers from SASTRA Deemed University in Thanjavur, Tamilnadu, provided data for the dataset. This dataset is built using 1036 word-level images, 700 fully annotated videos with a large vocabulary, and 18,863 sentence-level frames. To make this dataset publicly accessible, it is organized based on signer variants and time boundaries. Converting a spoken sentence into sign languages is the primary goal, and difficulties are resolved to improve recognition performance.

These datasets are available via: https://data.mendeley.com/datasets/kcmpdxky7p/1#:~:text=The%20ISL%2DCSLTR%20corpus%20consists,performed%20by%207%20different%20Signers, accessed on 2 December 2024.

In addition to our proposed ISL-CSLTR dataset, we also assessed the model’s performance on a publicly available American Sign Language (ASL) dataset (WLASL-2000 Resized, available on https://www.kaggle.com, accessed on 2 December 2024) to establish a comparative benchmark and confirm the model’s robustness. To demonstrate how well our dataset and framework recognize dynamic sign language across various linguistic domains, this comparison is shown in Table 3.

### 4.4. Performance Metrics

A range of performance metrics are leveraged to estimate the performance of the suggested approach for double-handed SL recognition. The False Negatives ($DSL_{fne}$), True Negatives ($DSL_{tne}$), False Positives ($DSL_{fps}$), and True Positives ($DSL_{tps}$) serve to measure efficiency parameters. False negatives are defined as expected instances that are incorrectly identified, true negatives as correctly identified, false positives as falsely identified double-handed SLs, and true positives as correctly identified double-handed SLs. The following are the performance measures’ definitions and formulas:

#### 4.4.1. Recognition Accuracy ($RA_{DSL}$)

The recognition accuracy is defined as follows, and this metric calculates the proportion of correctly identified dual-handed sign language samples to the total number of identified dual-handed sign language samples:

(28) $$RA_{DSL}=\frac{DSL_{tps}+DSL_{tne}}{DSL_{tps}+DSL_{fps}+DSL_{tne}+DSL_{fne}},$$

#### 4.4.2. Precision ($PR_{DSL}$)

This assesses the framework’s accuracy in identifying the double-handed SL samples and is defined as

(29) $$PR_{DSL}=\frac{DSL_{tps}}{DSL_{tps}+DSL_{fps}},$$

#### 4.4.3. Recall ($RC_{DSL}$)

The relative amount of correctly identified dual-handed sign language samples to the sum of dual-handed sign language samples in the dataset is known as recall, and it is calculated as

(30) $$RC_{DSL}=\frac{DSL_{tps}}{DSL_{tps}+DSL_{fne}},$$

#### 4.4.4. F1-Score ($FS_{DSL}$)

This is evaluated using the equation shown below and measures the combined effectiveness of precision and recall.

(31) $$FS_{DSL}=\frac{2*DSL_{tps}}{2*DSL_{tps}+DSL_{fps}+DSL_{fne}},$$

#### 4.4.5. AUC-ROC

This evaluates how well the framework distinguishes between a double-handed SL sample that is recognized and one that is not. Both the detection rate and the error rate are used to compute the discriminator curve. The method is more efficient when the AUC-ROC value is higher.

### 4.5. Performance Analysis

The effectiveness of the suggested approach in dual-handed sign language recognition is confirmed through efficiency evaluation. Table 3 displays the experimental analysis obtained using the suggested approach. This table makes it abundantly evident that the suggested approach obtained improved efficiency metrics, demonstrating its superiority in identifying dual hand sign language.

The performance outcomes of the suggested method on the ISL-CSLTR dataset and the proposed dataset are shown in Table 4. Here, the suggested method’s performances on the ASL dataset and the proposed dataset are evaluated using a number of performance metrics. The table guarantees that the suggested approach outperformed the ASL dataset in terms of performance values on the proposed dataset.

The interpretation process result and attention map generated by the suggested framework in dual-handed sign language identification using the proposed dataset are visualized in Table 5 and Table 6. The hand gesture is shown graphically as an attention map, and the table lists the dynamic words and the related input images that were collected from the video frames. It demonstrates unequivocally how the suggested method correctly identified the double-handed SL in the input images. Additionally, the area that corresponded to dual-handed signing patterns for the hearing-impaired received a lot of attention according to attention maps.

**Figure 3:**
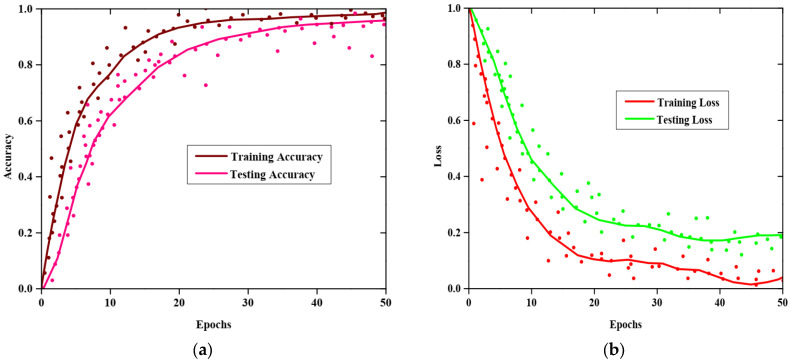
Evaluation of (**a**) accuracy, (**b**) loss.

Figure 3a,b display the accuracy and error rates derived from the suggested approach during training and validation stages. In this case, with more epochs, the accuracy level rises and the error value falls. The suggested approach obtained training and testing accuracies which were observed to be 0.9658 and 0.9488 at epoch 50, as shown in Figure 3. Additionally, the suggested method’s training and testing losses at the 50th epoch are 0.04 and 0.19, respectively, in Figure 3b. To put it another way, those numbers demonstrate that the suggested approach achieved both enhanced accuracy and reduced loss.

**Figure 4:**
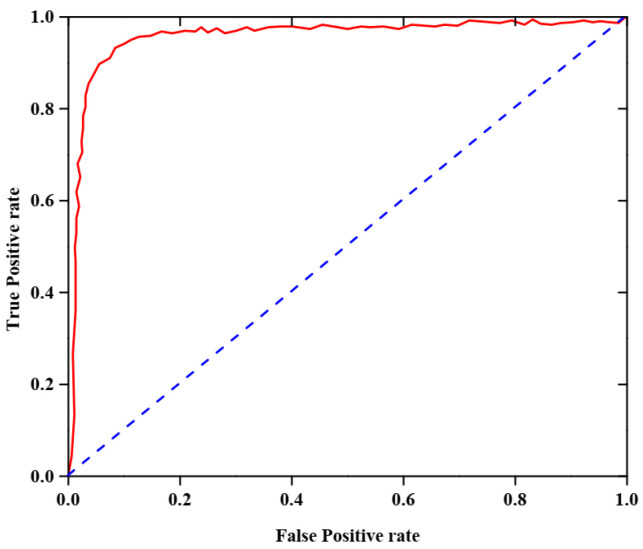
AUC-ROC analysis.

The AUC-ROC analysis of the proposed dataset is shown in Figure 4. This figure, which focuses on the reliability of the developed model in identifying valid and invalid dual-handed sign language movements, highlights an AUC-ROC rate of 0.988 for the proposed dataset.

### 4.6. Comparative Evaluation

The suggested approach is contrasted with cutting-edge techniques like Meta-ELM [12], SVM-CNN [17], LSTMRNN-KNN [18], T-CNN [19], TDDRMN [25], and PSO-CNN [27] in this subsection. Below is a detailed description of the comparative study’s findings.

**Figure 5:**
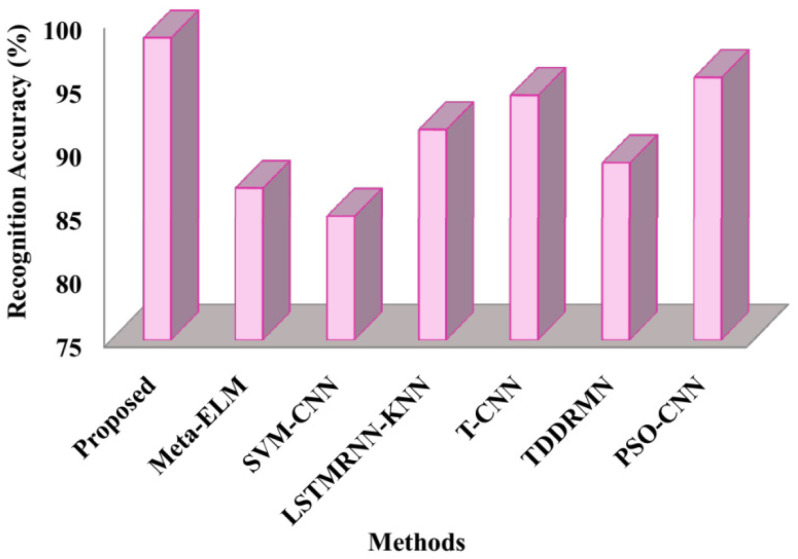
Comparison of recognition accuracy.

The measured recognition accuracy rates of the suggested and cutting-edge methods are displayed in Figure 5. The figure depicts that the suggested framework had a recognition accuracy of 98.69%, whereas it shows the corresponding accuracies of Meta-ELM as86.9%, SVM-CNN as 84.7%, LSTMRNN-KNN as 91.5%, T-CNN as 94.2%, TDDRMN as 88.9%, and PSO-CNN as 95.6%. According to the results, the suggested method performs better for double-handed SL recognition than the compared approaches because it has higher recognition accuracy.

**Figure 6:**
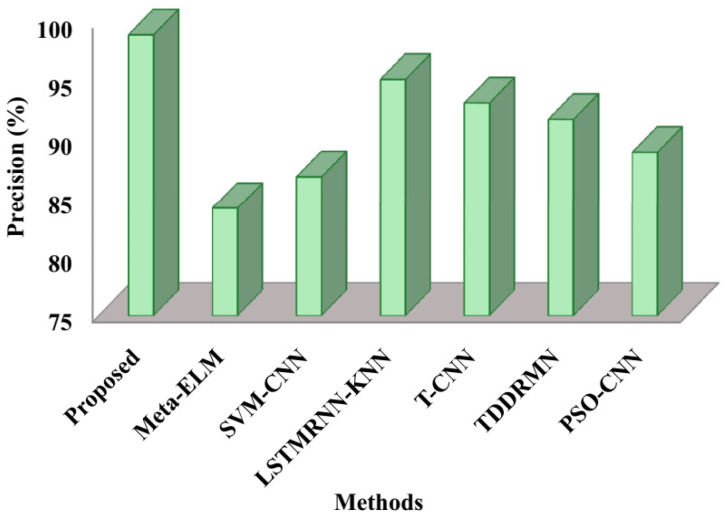
Comparative analysis of precision.

Figure 6 elaborates on the precision comparison. With Meta-ELM achieving scores of 84.1%, SVM-CNN 86.8%, LSTMRNN-KNN 95.1%, T-CNN 93.1%, TDDRMN91.7%, and PSO-CNN 88.9%, respectively, the recommended method achieved a precision of 98.9%. Therefore, the ability of the suggested approach is demonstrated by the above figure, which shows how well the double-handed SLs are recognized for the hearing-impaired.

**Figure 7:**
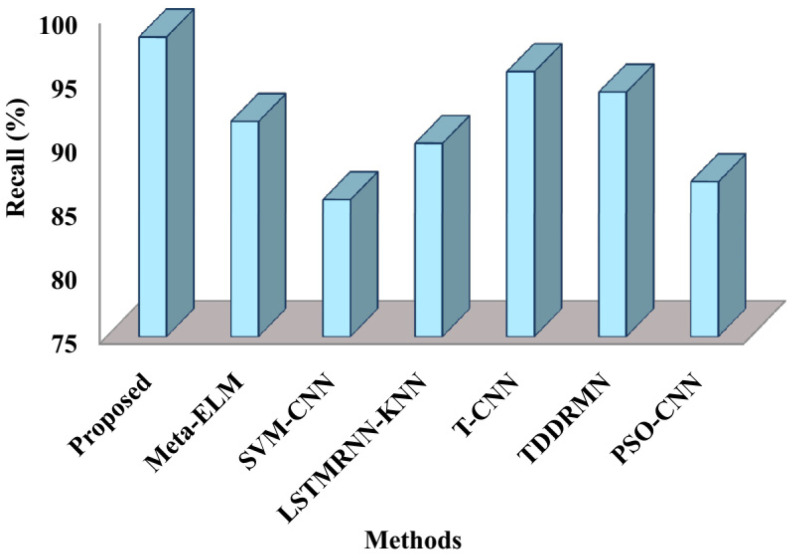
Comparison of recall.

The recall comparison between the suggested and cutting-edge methods is displayed in Figure 7. As shown in the figure, the proposed method recorded a recall of 98.4%, with Meta-ELM yielding recall values of 91.8%, SVM-CNN 85.7%, LSTMRNN-KNN 90.1%, T-CNN 95.7%, TDDRMN 94.1%, and PSO-CNN 87.1%, respectively. This comparison shows that the suggested approach has a high recall value and has successfully identified double-handed SL.

**Figure 8:**
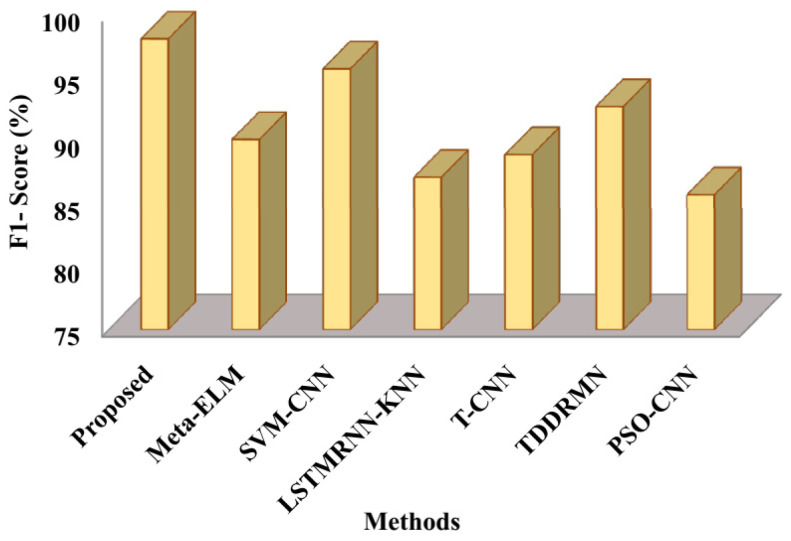
Comparative analysis of F1-score.

In terms of F1-score, Figure 8 contrasts the suggested and cutting-edge methods. The evaluated approaches yielded the following F1-scores: the suggested approach achieved 98.08%, Meta-ELM 90.1%, SVM-CNN 95.7%, LSTMRNN-KNN 87.1%, T-CNN 88.9%, and TDDRMN 92.7%, compared to PSO-CNN with85.7%. The aforementioned figures make it evident that the suggested method outperforms the compared approaches in terms of F1-score, highlighting its efficacy in identifying dual-handed sign language.

**Figure 9:**
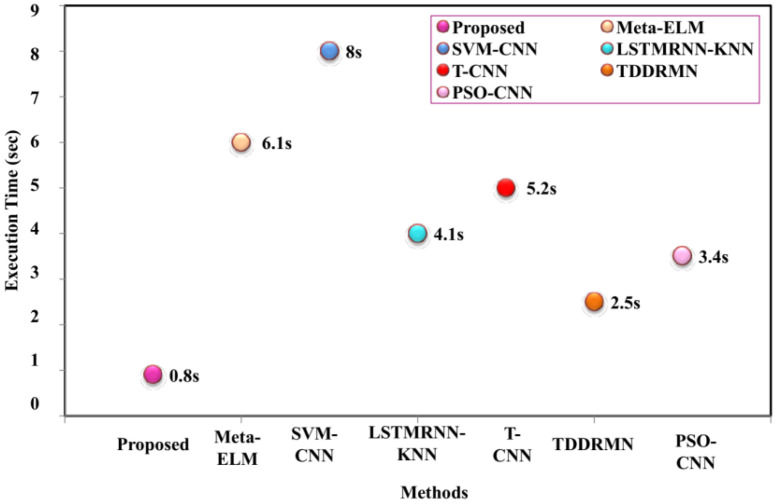
Analysis of execution time.

**Figure 10:**
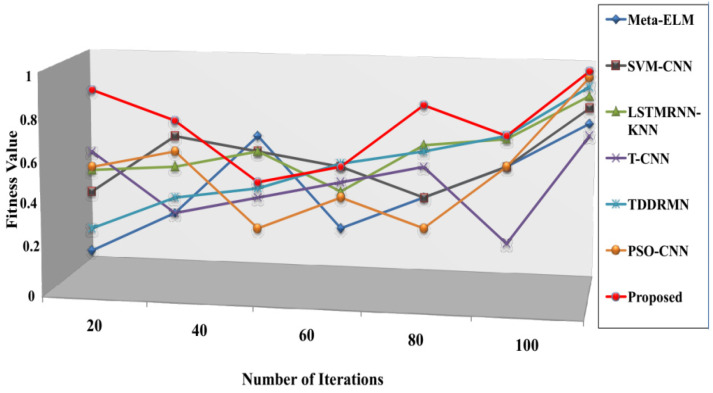
Comparison of convergence speed.

The comparison runtime analysis, which is the amount of time needed for the method to finish running, is shown in Figure 9. A shorter execution time indicates that the method is more efficient. Figure 10 illustrates that the proposed method takes 0.8 s to execute, compared to Meta-ELM 6.1, SVM-CNN 8, LSTMRNN-KNN 4.1, T-CNN 5.2, TDDRMN 2.5, and PSO-CNN 3.4 s, respectively.

The speed at which it approaches the ideal solution, shown in Figure 10, is known as the convergence rate. These results therefore suggest that the suggested approach executes faster than alternative approaches. The respective fitness values of the suggested approach 0.987, Meta-ELM 0.84, SVM-CNN 0.87, LSTMRNN-KNN 0.90, T-CNN 0.81, TDDRMN 0.92, and PSO-CNN 0.94 are shown in this figure. Lower figures illustrate that the suggested approach would have a higher fitness value than the approaches that are highlighted, highlighting the ATSO algorithm’s quick convergence speed in locating the best answers. To ensure a fair and consistent analysis of algorithmic performance, all of the methods compared in Figure 5, Figure 6, Figure 7, Figure 8, Figure 9 and Figure 10 were implemented and assessed on the same ISL-CSLTR dataset.

### 4.7. Ablation Study

Ablation experiments were carried out by gradually deleting or swapping out the following modules in order to evaluate the distinct contributions of the essential elements in the suggested ECT-ATSO framework:

- ATO Optimization;
- CNN Module;
- IRFFN Module; and
- Grain Module (GM).

In the experiments, the ISL-CSLTR dataset was used, and performance was assessed using execution time, F1-score, recall, accuracy, and precision. Table 7 presents the results of the ablation experiments:

Removing the CNN or Grain Modules dramatically reduces performance, as Table 6 demonstrates, demonstrating their vital role in improving local and texture-specific feature extraction. Likewise, in multi-class sign recognition, the IRFFN Module helps to increase classification precision and accuracy.

All metrics showed appreciable performance declines in the absence of ATSO optimization, especially convergence speed and overall accuracy, confirming the optimizer’s function in overcoming local optima and optimizing the hyperparameters. These results reaffirm that each module meaningfully enhances the overall effectiveness of the proposed ECT-ATSO framework.

These ablation study results clearly demonstrate the necessity and effectiveness of the Grain Module, CNN Module, IRFFN Module, and ATSO optimization in achieving superior recognition accuracy and model convergence performance for dual-handed Indian Sign Language recognition.

### 4.8. Convergence and Complexity Analysis

#### 4.8.1. Convergence Analysis

By examining the fitness value progression over training iterations, the suggested ECT-ATSO framework’s convergence behavior was assessed. The model quickly converges within the first 100 iterations, stabilizing at a fitness value of 0.987, as shown in Figure 10. In contrast to conventional gradient-based techniques, this illustrates how well the Adaptive Tuna Swarm Optimization (ATSO) algorithm accelerates convergence and avoids local optima.

#### 4.8.2. Computational Complexity

The Vision Transformer module, which necessitates O(N^2^) operations for self-attention, where N is the number of image patches, is the main source of the ECT-ATSO model’s computational complexity. Nonetheless, the overall model is still computationally viable for real-time applications by restricting the patch size and embedding dimension. On a typical CPU configuration (Intel Core i3-1005G1, 8GB RAM), the model achieves an inference time of about 0.8 s per frame and has roughly twenty [insert exact number] trainable parameters. This shows that the model maintains a realistic execution speed even with the additional transformer layers.

#### 4.8.3. Robustness to Variations in Data Quality

In order to evaluate robustness, a number of comprehensive preprocessing techniques were used, including data augmentation, Z-score normalization, and median filter denoising. These methods enhance the model’s capacity to manage input images with varying lighting, low contrast, and noise. Empirically, the model demonstrated strong resilience to changes in data quality by maintaining high recognition accuracy even when tested on augmented samples that replicated real-world degradations.

## 5. Conclusions

In order to identify dual-handed sign language for hearing-impaired people, this study presents a novel ECT-ATSO method. The ISL-CSLTR dataset is used as the source of the input images for this study, and this dataset is used to build the proposed dataset. The proposed dataset’s images are first preprocessed using a variety of preprocessing techniques to enhance their quality and generalization capabilities. These previously processed photos are then augmented with a grain pattern by the grain model. Extraction of key features is carried out using the CViT model. CNN and ViT transformer blocks make up this model. While the ViT transformer retrieves the comprehensive features extracted from the grained model, the CNN retrieves the local attributes. The identified local and global features are then integrated in the aggregation layer to create the feature representation.

The derived feature map is then sent to the categorization stage. During this stage, the IRFFN model is used to generate variable sign language lexicon generated from the feature map. Furthermore, outputs for multi-class classification are produced. Additionally, the CViT model’s parameters are adjusted using the ATSO algorithm to obtain the best result. Extensive experiments are conducted to assess the suggested method’s performance using industry-standard performance metrics. According to the experimental results, the suggested method performed better than the state-of-the-art techniques, achieving an F1-score of 98.08%, recall of 98.4%, precision of 98.9%, and a recognition accuracy of 98.69%.

These findings illustrate the success of the developed framework in identifying dual-handed sign language, guaranteeing that it is a useful application for people with hearing and communication challenges to interact with the general public. More datasets and sophisticated techniques will be added to the suggested approach in the future to boost the number of dynamic words produced for double-handed SL. Furthermore, this study can incorporate the advanced hand pose evaluation phase to optimize the suggested method’s recognition performance for intricate dual hand sign language images.

Furthermore, it is acknowledged that the combined training of transformer-based architectures with metaheuristic optimization algorithms may result in additional computational overhead during the training phase, even though the suggested ECT-ATSO framework achieves superior recognition accuracy and convergence speed. Further work will concentrate on lowering model complexity for large-scale, real-time applications, even though the ATSO optimization is limited to hyperparameter tuning in training and has no effect on inference-time latency. To further increase computational efficiency without sacrificing recognition accuracy, future improvements might incorporate lightweight transformer architectures like MobileViT or Linformer and employ distributed or parallelized optimization techniques.

The integration of skeleton-based hand pose estimation techniques for dual-handed sign language recognition will be investigated in future research, along with maximizing the computational efficiency of the suggested framework. Hand joint coordinates could be extracted as compact, interpretable features using techniques like OpenPose, MediaPipe Hands, or custom keypoint-based models, which could lessen the computational load and input dimensionality. Particularly in complex and cluttered backgrounds, combining transformer-based architectures with skeleton-based features may improve recognition accuracy even more while preserving real-time performance.

## Figures and Tables

**Table 1:** Research gaps and related contributions.

Identified Gap	Limitation in Existing Work	How This Work Addresses It
Limited focus on dynamic two-handed Indian Sign Language (ISL)	Most previous works concentrate on static gestures or American Sign Language (ASL), with little attention to the unique structure and complexity of dynamic two-handed ISL.	This work develops and evaluates a recognition system specifically tailored for dynamic two-handed ISL gestures.
Inadequate modeling of complex gesture dynamics	Traditional models like CNNs capture only local features, while RNNs often fail to learn long-range spatial and temporal dependencies needed for continuous gesture understanding.	The proposed hybrid approach combines CNNs for local feature extraction with Vision Transformers to learn global spatial relationships and temporal patterns effectively.
Suboptimal training and convergence performance	Many deep learning models rely solely on gradient-based optimization, which may converge slowly or get trapped in local minima.	This study integrates an Adaptive Tuna Swarm Optimization (ATSO) algorithm to improve convergence speed and optimize network parameters more effectively.
Sensitivity to variations in gesture input quality	Existing systems often show reduced accuracy under inconsistent lighting, complex backgrounds, or signer variability.	The framework incorporates robust preprocessing techniques (denoising, normalization) and data augmentation to enhance resilience to noise and variability.
Lack of evaluation for real-time feasibility	Few studies provide insights into computational cost, runtime performance, or suitability for real-time deployment.	This study includes a detailed analysis of model complexity, runtime efficiency, and inference time to assess practical deployment potential.

**Table 2:** Hyperparameter configurations.

Algorithms	Hyperparameter	Measurements
ECT	BatchSize	36
	FilterSize	[1 × 1, 3 × 3]
	Activation Function	Softmax
	LearningRate	0.0001
	DropoutRate	0.3
ATSO	m	0.05
	PopulationSize	50
	Aggregate iteration count	500
	j	0.6
	MutationRate	0.06

**Table 3:** Experimental analysis of the suggested framework.

Evaluation Metrics	Outcome/Value
Overall Accuracy	98.69%
Recall (Sensitivity)	98.4%
Precision (Positive Predictive Value)	98.9%
F1-Score	98.08%
Execution Duration	0.8 s
AUC-ROC (Area Under the ROC Curve)	0.988

**Table 4:** Experimental outcomes for datasets.

Datasets	Success Rate (%)	Sensitivity (%)	Specificity (%)	F-Measure (%)
ASL Dataset	92.5%	91.3%	93.4%	90.6%
Proposed Dataset	98.69%	98.4%	98.9%	98.08%

**Table 5:** Word-level identification findings and attention maps generated by the suggested approach making use of the proposed dataset.

Words	Original Images	Attention Maps
Angry	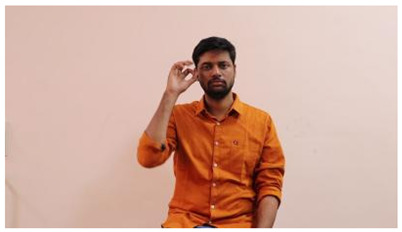	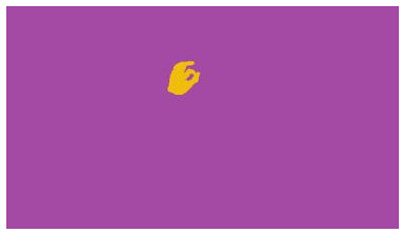
Afraid	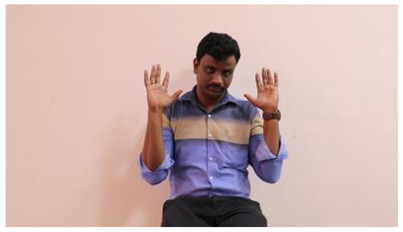	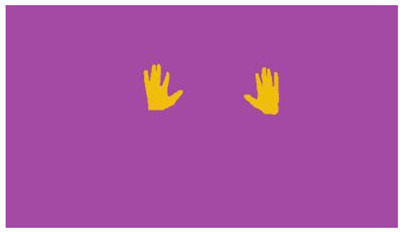
Appreciate	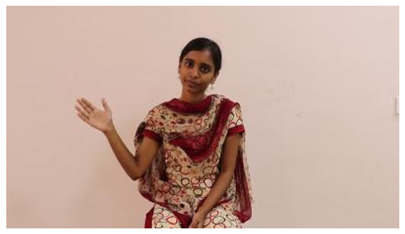	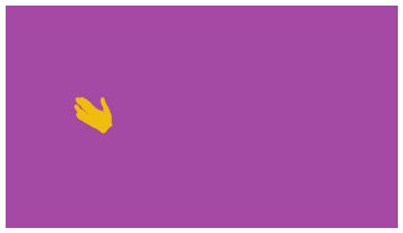
Bad	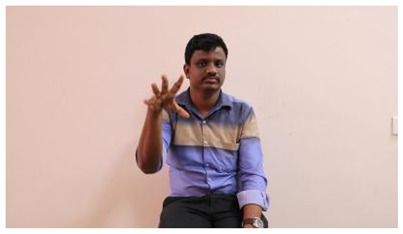	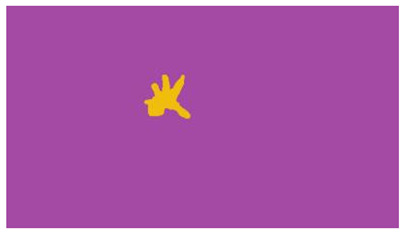
Bored	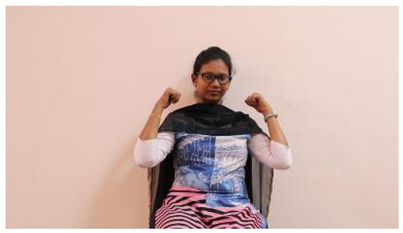	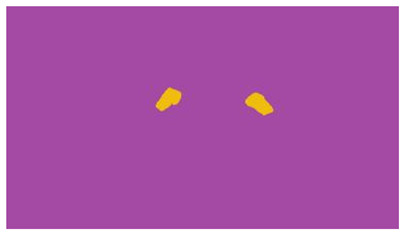
College or School	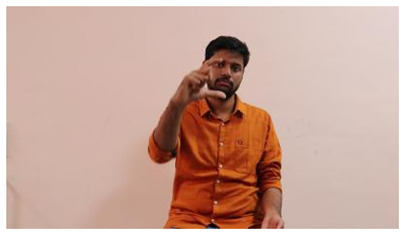	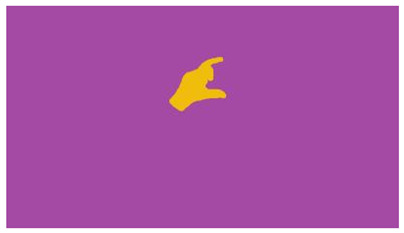
Dilemma	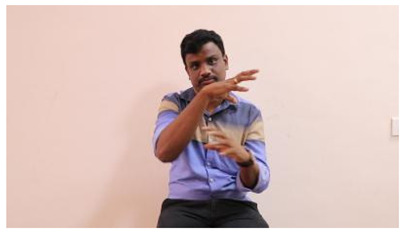	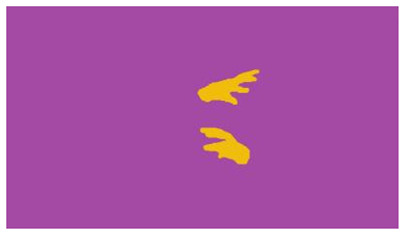
Don’t care	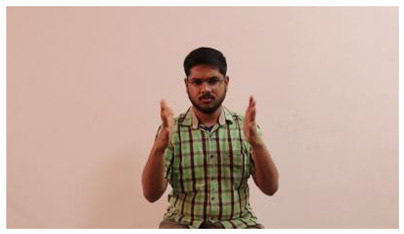	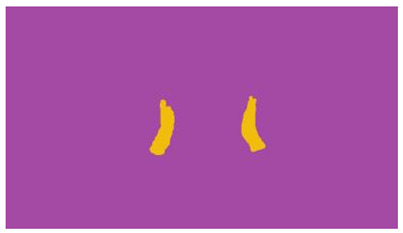
Grateful	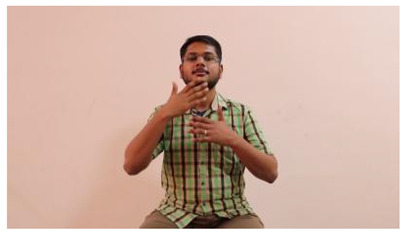	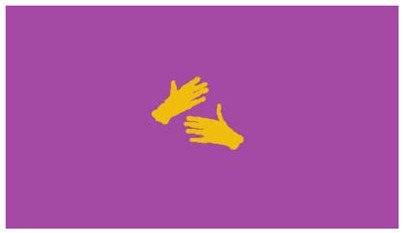
Hi or Hello	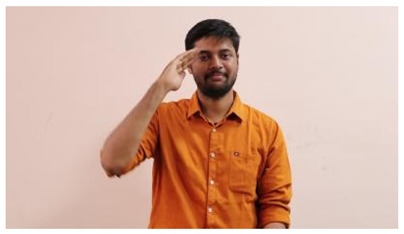	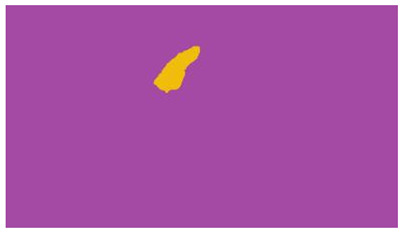

**Table 6:** Sentence-level identification findings and attention maps generated by the suggested approach making use of the proposed dataset.

Sentences	Frames and Corresponding Attention Map			
	Original Images		Attention Maps	
Bring water for me	* 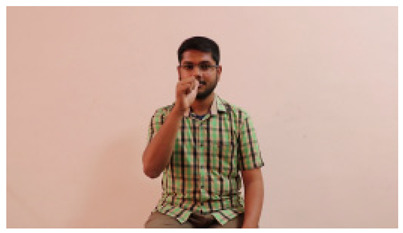 *	* 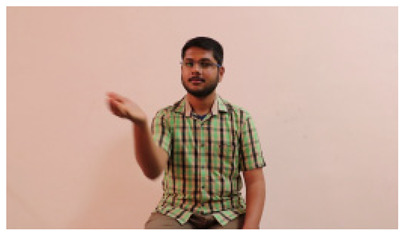 *	* 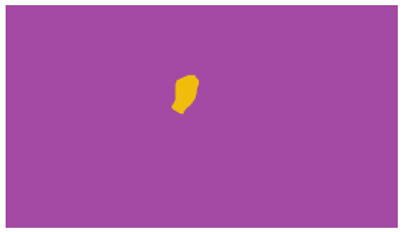 *	* 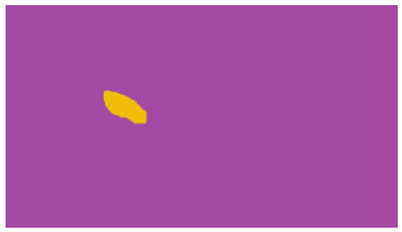 *
	* 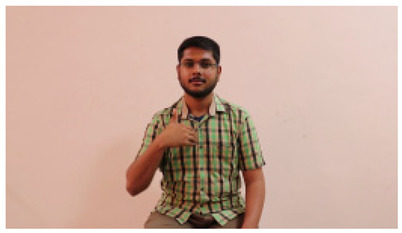 *		* 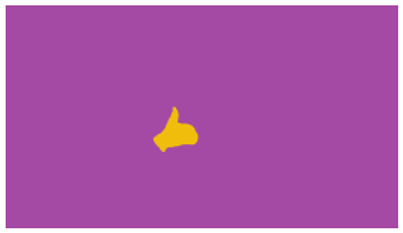 *	
Can I help you?	* 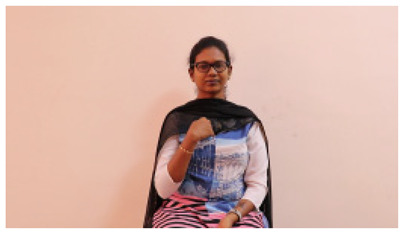 *	* 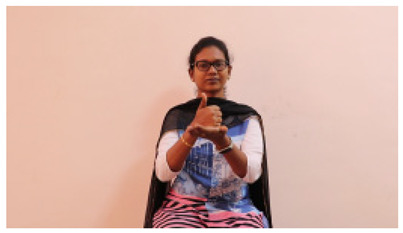 *	* 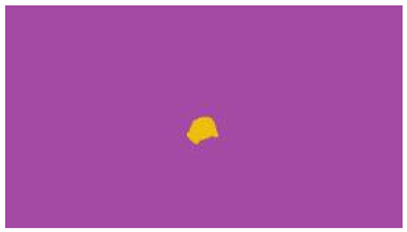 *	* 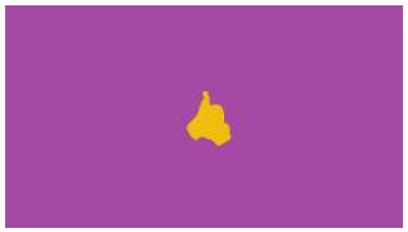 *
	* 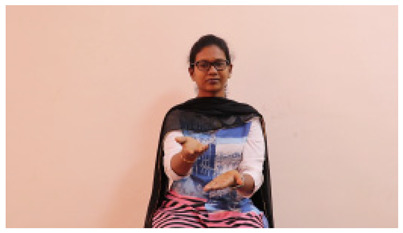 *		* 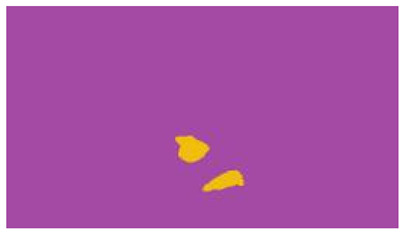 *	
Could you please talk slower?	* 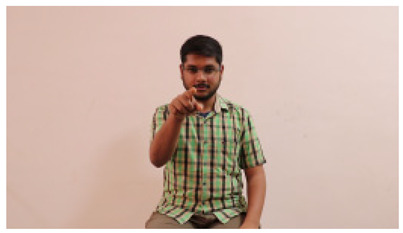 *	* 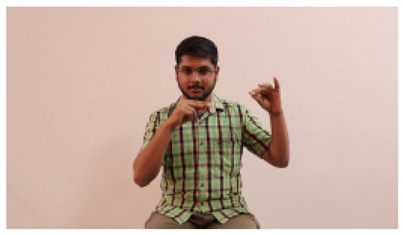 *	* 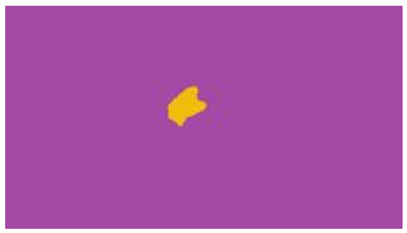 *	* 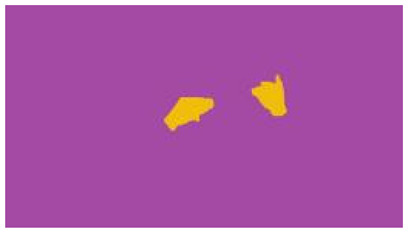 *
	* 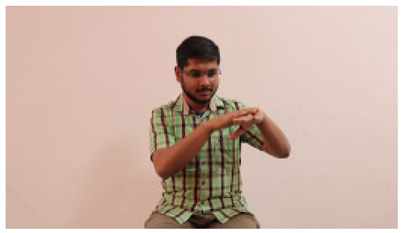 *		* 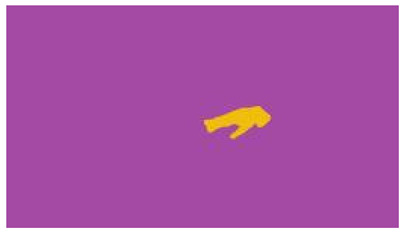 *	
Do not abuse him	* 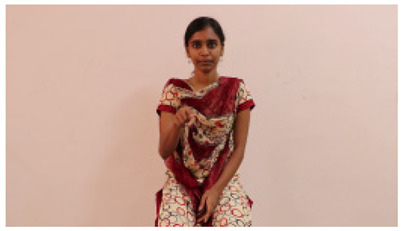 *	* 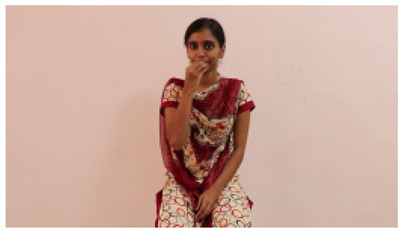 *	* 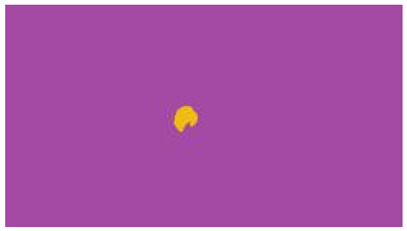 *	* 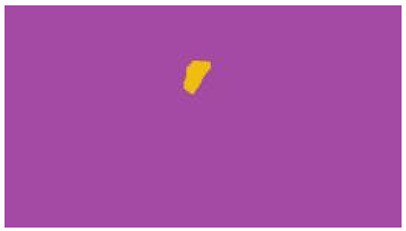 *
	* 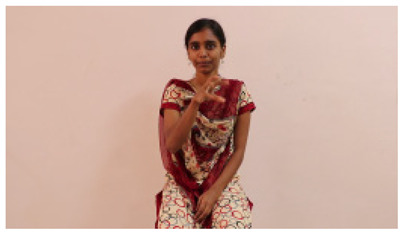 *	* 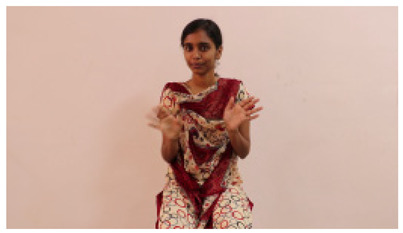 *	* 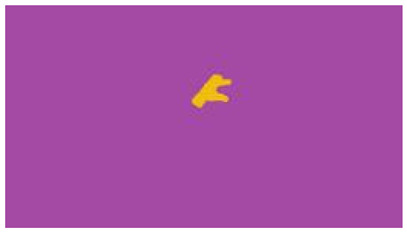 *	* 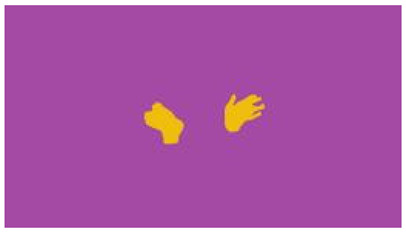 *
	* 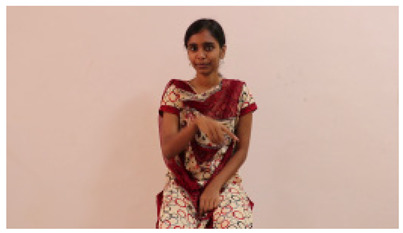 *		* 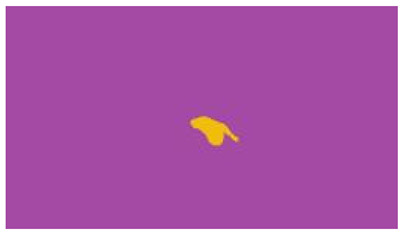 *	
Do not hurt me	* 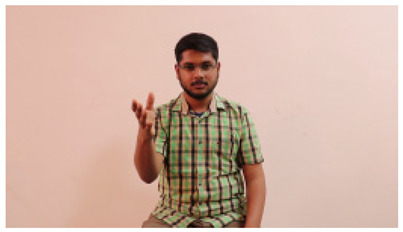 *	* 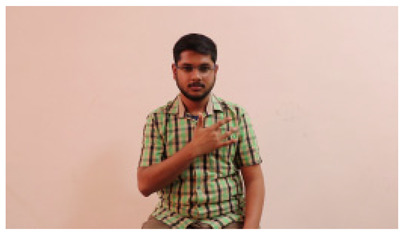 *	* 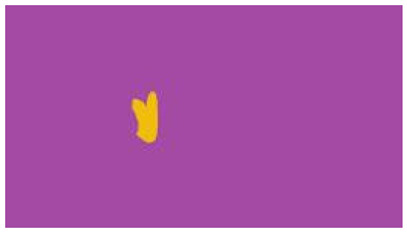 *	* 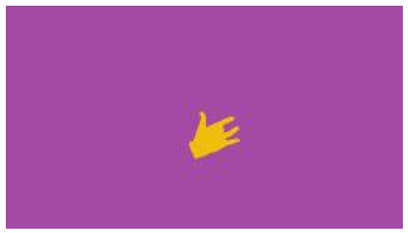 *
	* 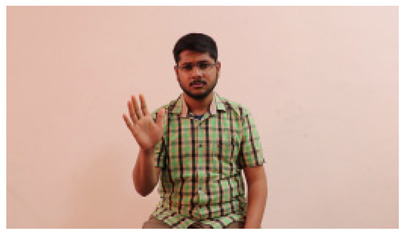 *		* 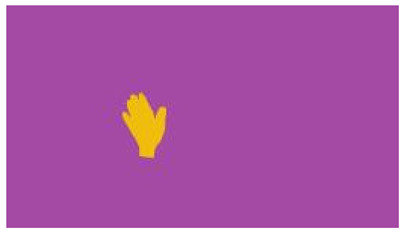 *	
He/She is my friend	* 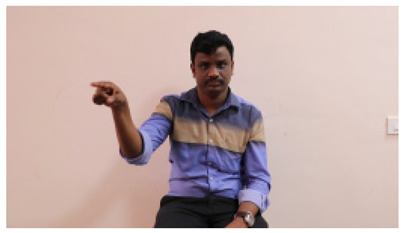 *	* 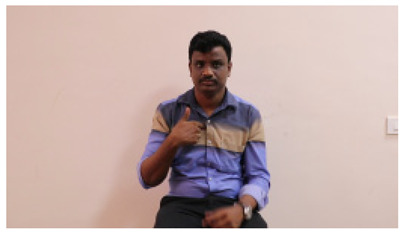 *	* 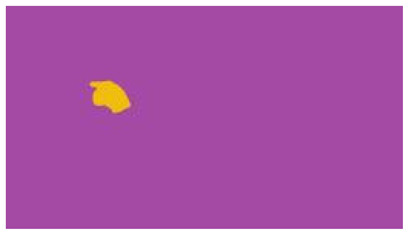 *	* 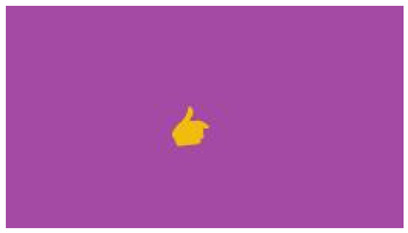 *
	* 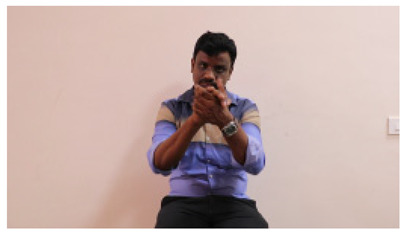 *		* 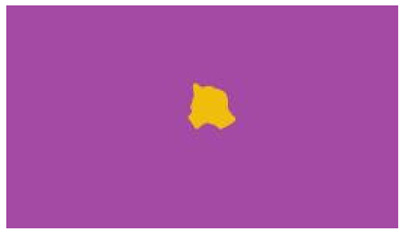 *	
How old are you?	* 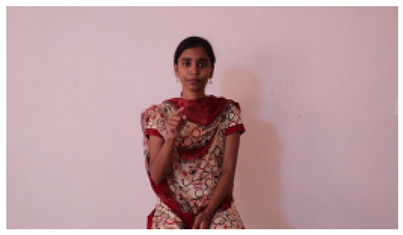 *	* 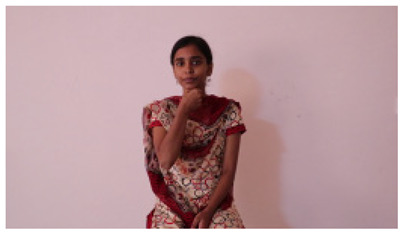 *	* 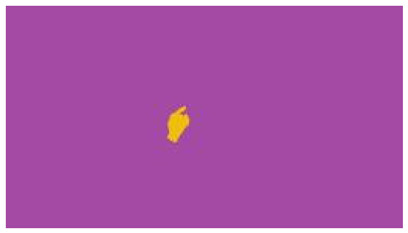 *	* 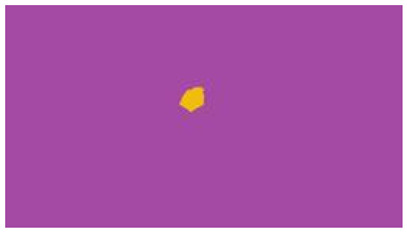 *
	* 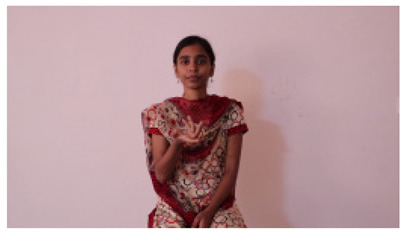 *		* 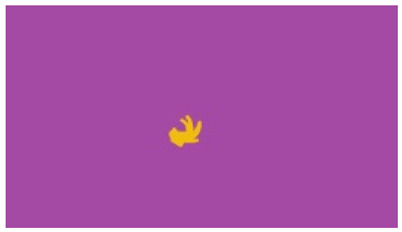 *	
I am afraid of that	* 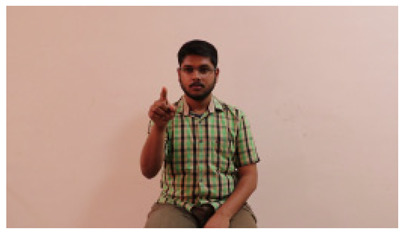 *	* 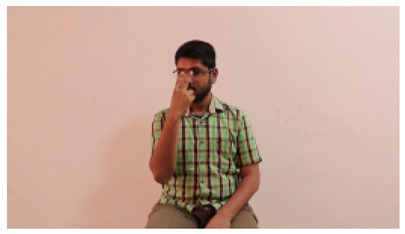 *	* 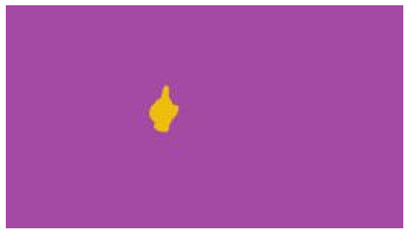 *	* 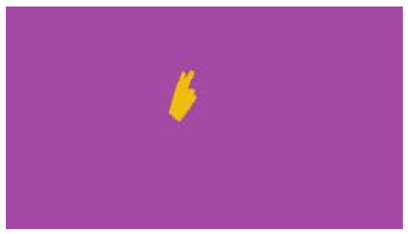 *
	* 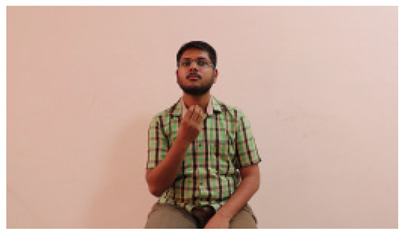 *	* 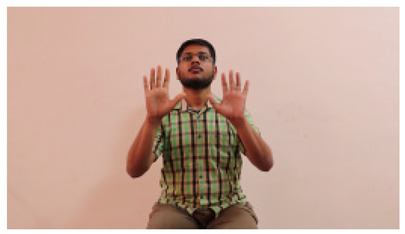 *	* 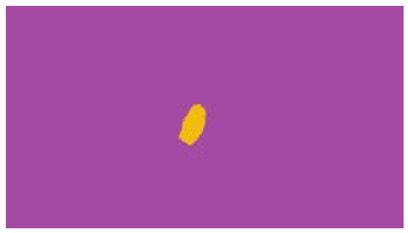 *	* 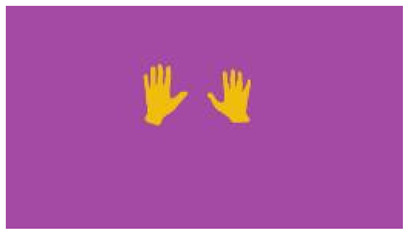 *
I am fine thank you	* 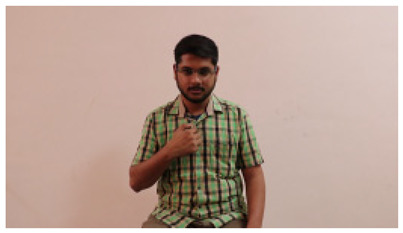 *	* 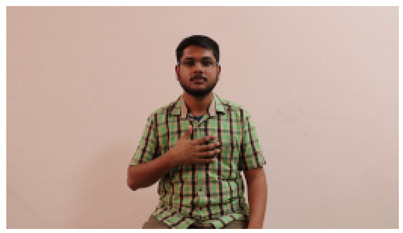 *	* 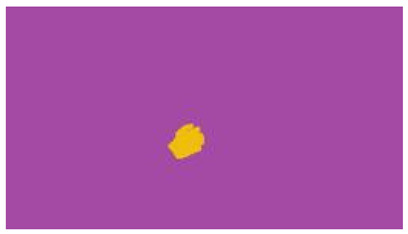 *	* 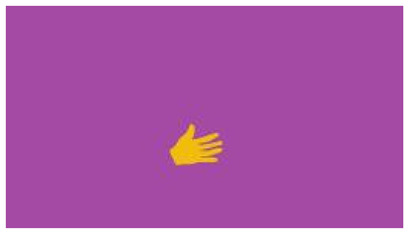 *
	* 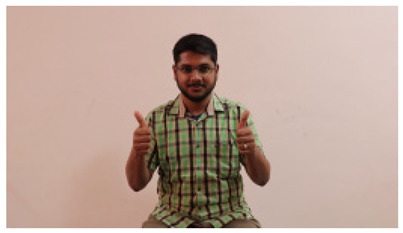 *	* 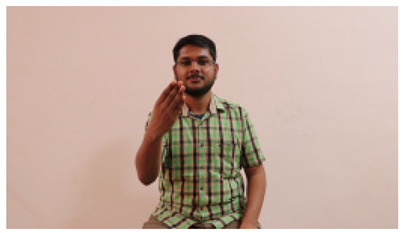 *	* 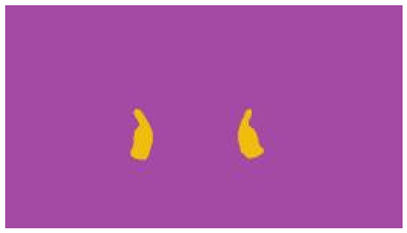 *	* 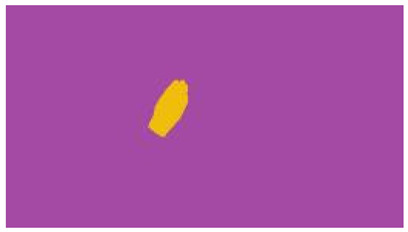 *
I am really grateful	* 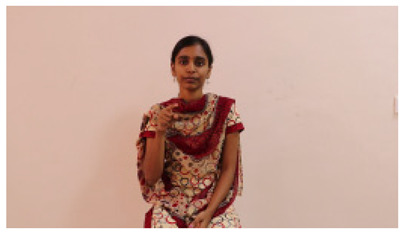 *	* 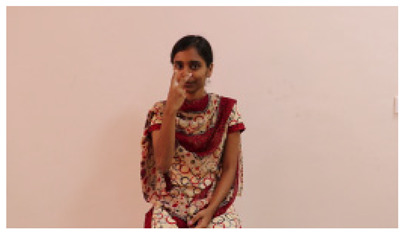 *	* 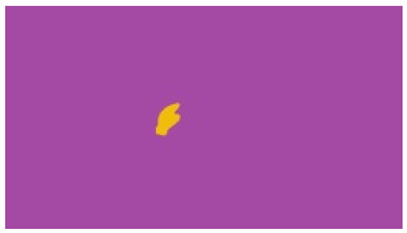 *	* 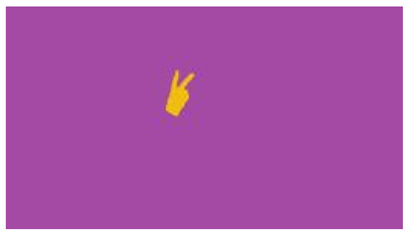 *
	* 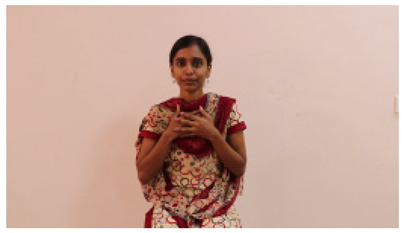 *		* 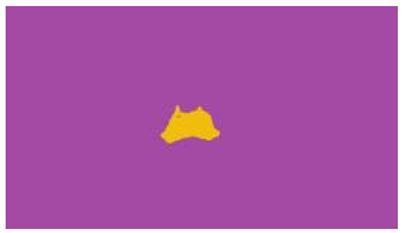 *	

**Table 7:** Ablation study results.

Configuration	Accuracy (%)	Precision (%)	Recall (%)	F1-Score (%)	Execution Time (s)
Full ECT-ATSO Framework	98.69	98.9	98.4	98.08	0.8
w/o Grain Module	96.55	96.8	96.1	96.3	0.72
w/o CNN Module	95.98	96.2	95.7	95.4	0.76
w/o IRFFN Module	96.87	97.1	96.4	96.6	0.79
w/o ATSO Optimization	94.22	94.5	94.1	93.8	0.73

## Data Availability

The experimental data are available at https://data.mendeley.com/datasets/kcmpdxky7p/1#:~:text=The%20ISL%2DCSLTR%20corpus%20consists,performed%20by%207%20different%20Signers, accessed on 2 December 2024.
